# Dr. Justin C. Elliott

**Published:** 1915-06

**Authors:** 


**Dr. Justin C. Elliott,** Buffalo 1851, died in Ardmore, Pa.,

April 8, aged 87. He formerly practiced in Springville, N. Y. and New Brighton, Pa.

